# Integrative transcriptomic and lipidomic analysis of the effects of *Eucommia ulmoides* on growth performance and lipid metabolism in *Larimichthys crocea*

**DOI:** 10.3389/fcimb.2026.1890262

**Published:** 2026-09-30

**Authors:** Xuan Zhang, Xiangyu Guo, Kun Hu, Qinghui Ai, Liyue Chai, Chengwei Huang, Yuanyuan He, Ping Zhuang, Jianping Wang, Rongrong Ma

**Affiliations:** 1 School of Marine Sciences, Ningbo University, Ningbo, China; 2 College of Fisheries and Life Science, Shanghai Ocean University, Shanghai, China; 3 Fisheries College, Ocean University of China, Qingdao, China; 4 Ningbo Institute of Oceanography and Fisheries, Ningbo, China

**Keywords:** *Eucommia ulmoides*, growth, *Larimichthys crocea*, lipid, lipidomic, transcriptome

## Abstract

**Introduction:**

*Larimichthys crocea* is an economically important marine aquaculture species, and improving growth performance while maintaining lipid metabolic homeostasis is important for its sustainable production. This study investigated the effects of dietary *Eucommia ulmoides* supplementation on growth performance and lipid metabolism in *L. crocea* and explored the underlying molecular basis using integrated transcriptomic and lipidomic analyses.

**Methods:**

A 4-week feeding trial was conducted using a control diet and a diet supplemented with 0.3% *E. ulmoides*. Growth performance, plasma biochemical indices, hepatic lipid accumulation, transcriptomic profiles, and lipidomic profiles were evaluated. Integrated pathway analysis was further performed to identify metabolic pathways jointly represented in the transcriptomic and lipidomic datasets.

**Results:**

Dietary supplementation with 0.3% E. ulmoides significantly increased the specific growth rate (SGR) of *L. crocea* (*P* < 0.05) and reduced the feed conversion ratio (FCR; 1.38 in the *E. ulmoides* group versus 2.68 in the control group). Hepatic Oil Red O staining revealed reduced lipid staining in the *E. ulmoides* group, while plasma biochemical analyses showed decreased triglyceride levels and improved antioxidant status. Integrated transcriptomic and lipidomic analyses identified glycerolipid metabolism and glycerophospholipid metabolism as the principal pathways represented in both omics datasets. In glycerolipid metabolism, differentially expressed genes (DEGs) were mapped to reactions involving intermediate metabolites associated with neutral-lipid synthesis. In glycerophospholipid metabolism, DEGs were mapped to reactions involving phosphatidylglycerol (PG), phosphatidylcholine (PC), phosphatidylethanolamine (PE), and their precursors. Notably, pathway-level co-mapping showed that these two metabolic pathways converge at phosphatidic acid (PA; 1,2-diacyl-sn-glycerol-3-phosphate), suggesting a potential metabolic connection between neutral-lipid storage and membrane lipid remodeling.

**Discussion:**

These findings indicate that dietary *E. ulmoides* supplementation can improve growth performance and modulate lipid metabolism in *L. crocea*. The coordinated changes in glycerolipid and glycerophospholipid metabolism, potentially linked through PA, provide a molecular basis for the observed reduction in hepatic lipid accumulation and suggest that *E. ulmoides* may promote lipid metabolic homeostasis in *L. crocea*.

## 1 Introduction?>

*Larimichthys crocea* is one of the most important marine aquaculture species in China, ranking first in production among farmed marine fish (Ministry of Agriculture and Rural Affairs of the People's Republic of China et al., 2025). It is highly valued by consumers for its palatable taste and nutritional content. However, with the rapid development of intensive aquaculture, long-term feeding of high-fat diets and high-density farming practices have been reported to induce metabolic disorders in *L. crocea*, subsequently leading to stunted growth and increased disease susceptibility (Naiel et al., 2023; Wang et al., 2013). For example, high levels of dietary olive oil have been shown to reduce growth and antioxidant capacity while activating the p38 MAPK and JNK signaling pathways associated with inflammation in this species (Li et al., 2019). Moreover, high-fat diets have been found to disrupt lipid homeostasis by suppressing farnesoid X receptor (FXR) activity through ER-stress-mediated activation of the same MAPK pathways, further promoting hepatic lipid deposition and metabolic dysfunction in *L. crocea* (Du et al., 2021). Lipid overload impairs hepatic very-low-density lipoprotein (VLDL) secretion through an oxidative stress-mediated PKCδ-HNF4α-MTP signaling pathway, resulting in excessive triglyceride accumulation in hepatocytes of *L. crocea* (Fang et al., 2021). These findings suggest that high-fat feeding induces a complex network of lipid metabolic dysregulation involving inflammatory signaling, oxidative stress, and impaired lipid transport. These physiological challenges may contribute to higher feed usage and management inputs, potentially affecting production efficiency and profitability (Gou et al., 2023). Therefore, finding effective feed improvement strategies to enhance both the metabolic health and growth efficiency of *L. crocea* have become research hotspots in the field of aquaculture. Current solutions mainly focus on adjusting feed composition, reducing culture density, and using antibiotics (Verdal et al., 2018; Hoseinifar et al., 2024). However, these methods have many disadvantages in practical applications. Nutritional strategies such as optimizing amino acid profiles and nutrient balance have been proven effective in improving fish growth and feed efficiency, but they require precise formulations and high-quality ingredients, which was limited by the long durations. Reducing culture density can improve the growth environment of fish, but it limits the scale and yield of aquaculture production. The use of antibiotics can lead to drug resistance, which may affect water ecological balance and food safety. Therefore, as a natural and green product, Chinese herbal medicine has gradually attracted attention in aquaculture. Its rich active ingredients have potential effects on regulating fat metabolism, promoting digestion and absorption, and enhancing immunity (Cai et al., 2023; Pu et al., 2017). Plant-derived bioactive compounds may also exert antimicrobial effects against aquatic pathogens; for example, quercetin has been reported to disrupt biofilm formation and attenuate the virulence of Aeromonas hydrophila (Jayaraman et al., 2024).

*E. ulmoides* is a traditional plant with both medicinal and edible uses, rich in various bioactive components such as lignans, iridoids, phenylpropanoids, flavonoids, and polysaccharides (He et al., 2014; Huang et al., 2021; Wang et al., 2019). Existing research has indicated that *E. ulmoides* and its active components possess the potential to regulate lipid metabolism in fish. In *Ctenopharyngodon idellus*, dietary supplementation with *E. ulmoides* bark and leaves can reduce mesenteric fat and crude fat content in muscles and affect lipid metabolism-related signaling pathways such as the PPAR pathway and the mTOR pathway (Yang et al., 2022). In *Ictalurus punctatus*, the extract of *E. ulmoides* leaves can alleviate liver steatosis induced by high-fat diets and regulate the expression of genes related to lipid synthesis and decomposition (Zhang et al., 2022). Additionally, chlorogenic acid (CGA), as one of the main phenolic active components in *E. ulmoides* leaves, also exhibits lipid-regulating effects in various fish species. It can improve the lipid metabolism status of *Carassius auratus* (Jin et al., 2022), alleviate lipid metabolism disorders in *Micropterus salmoides* induced by high-fat diets (Yin et al., 2021), and reduce the degree of hyperlipidemia in *Lateolabrax maculatus*, improving liver histological characteristics and lipid metabolism status (Ma et al., 2023).

Overall, current research shows that *E. ulmoides* can influence lipid deposition, liver lipid homeostasis, and the expression of lipid metabolism-related genes in fish. However, these evidences are mainly based on blood lipid, biochemical indicators, histological phenotypes, or a limited number of lipid metabolism-related genes. There is still a lack of systematic research on whether *E. ulmoides* can reshape the lipid composition of marine fish at the lipid molecular level and the relationship between such changes and transcriptional regulation. In *L. crocea*, dietary supplementation with *E. ulmoides* leaf extract has been proven to promote growth, enhance digestive enzyme activity and antioxidant capacity, and regulate the expression of genes related to immunity and feeding (Huang et al., 2021). However, its impact on the lipid molecular profile and the association with transcriptional responses remain unclear. Traditional growth and plasma biochemical indicators can reflect the overall physiological status but are difficult to analyze the relationships among gene regulation, metabolic pathways, and specific lipid molecular changes. Transcriptomics can analyze biological responses at the gene and pathway levels, while lipidomics can directly characterize changes in lipid classes and molecular species. Therefore, integrating these two omics can provide complementary information at different levels (Eide et al., 2023) and contribute to establishing a “gene-pathway-lipid” association framework, thereby more systematically analyzing the regulatory effect of *E. ulmoides* on lipid metabolism in *L. crocea*.

Based on this, we hypothesize that the addition of *E. ulmoides* in feed can improve the growth performance and lipid metabolism status of *L. crocea*. To verify this hypothesis, we evaluated the growth performance and biochemical indicators, and characterized the relevant molecular responses through transcriptome and lipidome analyses. By jointly analyzing differentially expressed genes and differentially expressed lipid molecules, we aim to further identify the major lipid metabolic pathways and gene-lipid associations related to *E. ulmoides* treatment, thereby providing molecular-level evidence for its potential application as a functional feed additive in large yellow croaker aquaculture.

## 2 Materials and methods

All experimental procedures involving *L. crocea* were conducted in accordance with the governing regulation for the use of experimental animals in Zhejiang Province (Zhejiang Provincial Government Order No. 263) and the Experimental Animal Management Law of China, following applicable industry standards for aquatic animal handling.

### 2.1 Feeding experiment

*L. crocea* was reared in small cages at a fish farm located in Xiangshan, Zhejiang Province. A total of 3600 fish with an average body weight of 170.06 ± 9.87 g at initial selection were randomly assigned to six experimental cages (4.0 × 4.0 × 5.0 m) and acclimated to the basal diet for one week. After acclimation, the 28-day feeding trial was initiated; the initial body weights at the start of the feeding trial were 177.70 ± 7.38 g in the CON group and 179.00 ± 8.67 g in the EU group, as reported in **Table 1**. The basic feed for *L. crocea* was procured from Zhejiang Qiangpu Biotechnology Co., Ltd., China. For the experimental group (EU group), 0.3% *E. ulmoides* was added to the basic feed. The formula and composition of the feed used in this experiment are detailed in **Table 2**. The proximate composition previously reported for the identical basal formulation from the same manufacturer was 48.83% crude protein, 12.23% crude lipid, 11.03% crude ash, and 8.08% moisture (Zhu et al., 2025). The EU diet was formulated by replacing 0.3% high-gluten flour with 0.3% *E. ulmoides* bark; treatment-specific proximate composition and gross energy were not independently determined in the present experiment. In the EU group, *E. ulmoides* bark was dried, ground into powder using a high-speed grinder, and sieved through a 100-mesh sieve. Then, *E. ulmoides* was thoroughly mixed with the feed. Both the EU group and the control group (CON group, fed only the basal diet) were fed twice daily, in the morning and evening, at a feeding rate of 3% of body weight. Three cages were set up for each group. Throughout the trial, water quality parameters were maintained as follows: salinity, 28–33‰; temperature, 20–26 °C; and dissolved oxygen, approximately 5 mg/L. The feeding trial lasted for 28 days. Throughout the entire experimental period, fewer than 5 fish died in each cage, corresponding to a survival rate of > 99.3% in all cages. For growth-performance analyses, each cage was treated as the experimental unit (n = 3 cages per treatment).

**Table 1:** Growth performance of *L. crocea* (n = 3 cages per treatment).

Group	Initial weight (g)	Final weight (g)	WG (g)	SGR (%·day^−1^)	FCR	CF (%)	HSI (%)
CON	177.70 ± 7.38	233.75 ± 16.40	56.05 ± 3.02^a^	0.98 ± 0.05^a^	2.68 ± 0.25^a^	1.67 ± 0.08^a^	2.17 ± 0.52^a^
EU	179.00 ± 8.67	288.34 ± 13.96	109.34 ± 19.76^b^	1.71 ± 0.19^b^	1.38 ± 0.20^b^	1.46 ± 0.15^b^	1.66 ± 0.31^b^

CON represents the control group; EU represents the *E. ulmoides* group. Data are presented as mean ± SD based on three cage-level replicates per treatment. The 15 fish sampled from each cage were treated as subsamples, and individual-fish measurements were averaged within cage before analysis; FCR was calculated independently for each cage. The initial weight reported in **Table 1** refers to body weight measured at the start of the 28-day feeding trial after the one-week acclimation period. Values in the same column with different superscript letters differ significantly (*P* < 0.05).

**Table 2:** Formulation of feed (% dry weight) used for *L.crocea*.

Ingredients (% dry weight)	CON	EU
Fish meal	38.0	38.0
Chicken powder	5.0	5.0
Soy protein concentrate	9.0	9.0
Soybean meal	14.5	14.5
Corn protein powder	5.0	5.0
High gluten flour	21.1	20.8
Fish oil	2.0	2.0
Soybean oil	2.0	2.0
Ca(H_2_PO_4_)_2_	1.0	1.0
Lutein	0.3	0.3
Choline chloride	0.1	0.1
Vitamin premix	0.5	0.5
Mineral elements	1.5	1.5
*E. ulmoides*	0	0.3
Previously reported proximate composition of the identical basal formulation (%)		
Crude protein	48.83	ND
Crude lipid	12.23	ND
Crude ash	11.03	ND
Moisture	8.08	ND

CON group was basal diet; EU group was supplemented with 0.3% *E. ulmoides*. The CON proximate-composition values are those previously reported for the identical basal formulation from the same manufacturer (Zhu et al., 2025). ND, not determined in the present experiment; therefore, treatment-specific proximate composition and gross energy of the EU diet are not reported as measured values.

### 2.2 Sample collection

*L. crocea* were fasted for 24 h prior to sample collection. Before sampling, fishes were analyzed by immersion in aerated seawater containing 100 mg/L tricaine methanesulfonate (MS–222; 0.01%, w/v), which was freshly prepared and buffered with sodium bicarbonate to pH 7.0–7.4. Fishes were maintained in the anesthetic bath until loss of equilibrium and reduced response to external stimuli were observed, and sampling was then performed immediately to minimize handling stress. During sampling, 15 fishes were randomly selected from each cage, and growth parameters including body weight, body length, and body height were recorded. The body weight gain (WG), specific growth rate (SGR), feed conversion ratio (FCR), and condition factor (CF) were then calculated. Whole-blood samples were collected from the caudal vein using sterile 1 mL syringes and immediately transferred into sterile heparinized anticoagulant tubes. The tubes were gently inverted several times to ensure complete mixing of blood with the anticoagulant and then kept at 4 °C for 24 h before centrifugation. The plasma fraction was subsequently separated from the whole blood by centrifugation and stored at −80 °C for subsequent biochemical analysis. The liver was dissected and weighed for hepato-somatic index (HSI) calculation. Then, muscle samples were placed into sterile, enzyme-free cryotubes, immediately flash-frozen in liquid nitrogen, and transported back to the laboratory for storage at −80 °C. The 15 fish sampled from each cage were treated as subsamples rather than independent experimental units. For growth-related variables measured at the individual-fish level, measurements were averaged within each cage to obtain one cage-level replicate before statistical analysis. FCR was calculated independently for each cage from total feed intake and biomass gain. The relevant calculation formulas are presented in Equations 1–5:

(1) $$Body\;weight\;gain\;\left(WG,\;g\right)\;=\;W_{t}-W_{0}$$

(2) $$Specific\;growth\;rate\;(SGR,\;\%\cdot day^{-1})\;=\;100\;\times \;(Ln\;W_{t}-Ln\;W_{0})/t,$$

(3) $$Feed\;conversion\;ratio\left(FCR\right)\;=\;Total\;feed\;intake\;per\;cage/biomass\;gain\;per\;cage$$

(4) $$Condition\;factor\;\left(CF,\;\%\right)\;=\;\left(W_{t}/L_{t}L^{3}\right)\;\times \;100,$$

(5) $$Hepato‐somatic\;index\;\left(HSI,\;\%\right)\;=\;\left(liver\;wet\;weight/W_{t}\right)\;\times \;100,$$

Where W_0_ and W_t_ represent the initial and final body weights, respectively; t is experimental days; L_t_ is the final body length. For FCR, biomass gain refers to the increase in total fish biomass within each cage over the feeding period.

### 2.3 Biochemical index determination

Stored plasma samples obtained during the terminal sampling described in Section 2.2 were thawed on ice, and four biological samples were analyzed per group (n = 4). Preliminary dilution tests were performed with 0.9% saline according to the manufacturers’ instructions, and the appropriate dilution was used for the formal assays. Plasma triglyceride (TG), glutathione (GSH), superoxide dismutase (SOD), and malondialdehyde (MDA) were measured using commercial assay kits purchased from Nanjing Jiancheng Bioengineering Institute (Nanjing, China).

### 2.4 Oil Red O staining of liver

Liver samples collected at the end of the feeding trial were processed for frozen-section Oil Red O staining. After formation of a firm embedding block, the samples were sectioned at 5–10 μm using a cryostat, mounted on glass slides, and stored at −80 °C until staining. Before staining, frozen sections were equilibrated at room temperature for 15 min and rinsed twice with distilled water for 2 min each. The sections were then washed twice with 60% isopropanol for 2 min each, and excess liquid was removed. Sections were stained with Oil Red O working solution for 5 min and subsequently differentiated with 60% isopropanol under microscopic observation until appropriate staining contrast was obtained. After differentiation, the sections were immediately rinsed with distilled water and counterstained with 50% hematoxylin for 30 s. The sections were then washed with distilled water for 5 min, mounted with glycerol gelatin, examined under a light microscope, and photographed. Oil Red O-positive neutral lipid deposits were visualized as red staining.

### 2.5 Lipidome analysis

Untargeted lipidomics was performed on *L. crocea* muscle samples selected from the terminal sampling described in Section 2.2, with five biological samples per group (n = 5). Approximately 50 mg of frozen muscle tissue was extracted with 750 µL chloroform:methanol (2:1, v/v) in a 2-mL centrifuge tube. After vortexing for 30 s, samples were homogenized with steel beads at 50 Hz for 60 s twice and incubated on ice for 40 min. Subsequently, 190 µL ultrapure water was added, followed by vortexing for 30 s and incubation on ice for 10 min. Samples were centrifuged at 12,000 rpm for 5 min at room temperature, and 300 µL of the lower organic phase was collected. The remaining sample was re-extracted with 500 µL chloroform:methanol (2:1, v/v), and an additional 400 µL of the organic phase was collected after centrifugation under the same conditions. The combined organic extracts were evaporated to dryness under vacuum, reconstituted in 200 µL isopropanol, and filtered through a 0.22-µm PTFE membrane before liquid chromatography-mass spectrometry (LC-MS) analysis.

Chromatographic separation was performed using a Vanquish UHPLC system coupled to a Q Exactive mass spectrometer (Thermo Fisher Scientific, USA). Lipids were separated on an ACQUITY UPLC BEH C18 column (2.1 × 100 mm, 1.7 µm; Waters) maintained at 50 °C, with the autosampler held at 8 °C. The flow rate was 0.25 mL/min and the injection volume was 2 µL. Mobile phase A consisted of acetonitrile:water (60:40, v/v) containing 0.1% formic acid and 10 mM ammonium formate, whereas mobile phase B consisted of isopropanol:acetonitrile (90:10, v/v) containing the same additives. The gradient, expressed as the percentage of mobile phase A, was programmed as follows: 70–57% at 0–5 min, 57–50% at 5–5.1 min, 50–30% at 5.1–14 min, 30% at 14–14.1 min, 30–1% at 14.1–21 min, 1% at 21–24 min, 1–70% at 24–24.1 min, and 70% at 24.1–28 min.

Mass spectrometric detection was performed using electrospray ionization in both positive- and negative-ion modes. Spray voltages were 3.5 and 2.5 kV in positive and negative modes, respectively. Sheath gas and auxiliary gas were set to 30 and 10 arbitrary units, respectively, and the capillary temperature was 325 °C. Full-scan spectra were acquired over m/z 150–2,000 at a resolution of 35,000. Data-dependent MS/MS acquisition was performed using higher-energy collisional dissociation at a collision energy of 30 eV, together with dynamic exclusion to minimize redundant fragmentation. No exogenous lipid internal standard was specified in the analytical protocol; therefore, lipid abundances were evaluated on a relative rather than absolute quantitative basis.

Raw MS data were processed using LipidSearch software for peak processing, alignment, and lipid molecular-species annotation. After data normalization, orthogonal partial least-squares discriminant analysis (OPLS-DA) was used to evaluate group separation. Differential lipid molecules (DLMs) were defined as those with *P* < 0.05 and a variable importance in projection (VIP) score > 1. Lipid ontology and biological-function enrichment analyses were subsequently performed using the LION database.

### 2.6 Transcriptome analysis

For transcriptome analysis, three biological samples per group (n = 3) were selected from the terminal sampling pool described in Section 2.2, and total RNA was extracted using TRIzol reagent following the manufacturer’s protocol. RNA purity and concentration were assessed using a NanoDrop 2000 spectrophotometer (Thermo Scientific, USA), and RNA integrity was evaluated using an Agilent 2100 Bioanalyzer (Agilent Technologies, USA). Transcriptome libraries were constructed using the VAHTS Universal V10 RNA-seq Library Prep Kit (Premixed Version) according to the manufacturer’s instructions, and sequencing and primary analysis were performed by OE Biotech Co., Ltd. (Shanghai, China). The libraries were sequenced on an Illumina NovaSeq 6000 platform to generate 150-bp paired-end reads. Raw reads in FASTQ format were filtered using fastp (Chen et al., 2018), and the resulting clean reads were aligned to the reference genome (https://www.ncbi.nlm.nih.gov/datasets/genome/GCF_000972845.2/) using HISAT2 (Kim et al., 2019). Gene expression abundance was reported as FPKM values (Roberts et al., 2011), whereas gene-level read counts were obtained using HTSeq-count (Anders et al., 2015) and used for differential expression analysis. Principal component analysis and hierarchical clustering were performed in R (v3.2.0). Differentially expressed genes (DEGs) were identified using DESeq2 (Love et al., 2014) with q-value < 0.05 and foldchange > 2 or foldchange < 0.5. KEGG pathway enrichment analysis (Kanehisa et al., 2008) was performed using a hypergeometric distribution algorithm.

### 2.7 Verification of DEGs by RT-qPCR

The reliability of the RNA sequencing (RNA-seq) data was validated by real-time reverse transcription quantitative PCR (RT-qPCR), using *β-actin* as the internal reference gene. Four DEGs were selected from the pathways related to lipid metabolism in the KEGG pathway enrichment analysis. Primer sequences are listed in **Supplementary Table S1**. RT-qPCR validation was performed using three biological replicates per group, corresponding to the biological samples used for RNA-seq. For each target gene, each biological replicate was analyzed in three technical replicate reactions. The RT-qPCR reaction was performed in a 10 μL system containing 5 μL of 2× ChamQ Blue Universal SYBR qPCR Mix, 0.2 μL each of forward and reverse primers, 1 μL of cDNA, and 3.6 μL of ddH_2_O. The thermal cycling conditions were as follows: initial denaturation at 95 °C for 30 s, followed by 40 cycles of denaturation at 95 °C for 10 s and annealing/extension at 60 °C for 30 s. The relative expression levels of the target genes were calculated using the 2^–ΔΔCt^ method.

### 2.8 Integrated transcriptomic and lipidomic analysis

Pathway enrichment results from the transcriptomic and lipidomic datasets were cross-compared to identify metabolic pathways represented in both omics layers. DEGs and DLMs annotated to these overlapping pathways were then jointly mapped onto KEGG pathway diagrams to visualize their pathway-level relationships and shared metabolic intermediates. The integrated analysis was restricted to pathway-level co-mapping and was not used to infer direct statistical correlations or co-regulatory relationships between individual genes and lipid molecules.

### 2.9 Statistical methods

Data are presented as the mean ± standard deviation (SD). Differences in growth performance and plasma biochemical parameters between the experimental groups were evaluated using one-way analysis of variance (ANOVA). For growth-performance data reported in **Table 1**, the cage was the experimental unit (n = 3 cages per treatment). The 15 fish sampled from each cage were treated as subsamples; individual-fish measurements were averaged within each cage before statistical comparison. FCR was calculated independently for each cage from total feed intake and biomass gain. Accordingly, statistical inference for **Table 1** was based on three cage-level replicates per treatment rather than on individual fish. A *P* value < 0.05 was considered statistically significant. Statistical significance was denoted as *P* < 0.05, *P < 0.01, and **P < 0.001. Figures were generated using Origin 2021 software.

## 3 Results

### 3.1 Growth performance

The growth performance of *L. crocea* in each group was assessed. **Table 1** showed the effects of *E. ulmoides* on growth indicators of *L. crocea*, including the WG, SGR, HSI, and CF. Compared with the CON group, the WG and SGR in the EU group were significantly increased, indicating that *E. ulmoides* supplementation notably enhanced growth in *L. crocea*. The FCR in the EU group (1.38 ± 0.20) was significantly lower than that in the CON group (2.68 ± 0.25) (*P* < 0.05), indicating a substantial improvement in feed utilization efficiency following *E. ulmoides* supplementation. Additionally, the EU group exhibited significantly lower HSI and CF values, suggesting that *E. ulmoides* reduced fat deposition.

### 3.2 Biochemical indicators of plasma

Compared with the CON group, plasma TG and MDA levels were significantly lower in the EU group (*P* < 0.001 and *P* < 0.01, respectively), whereas SOD activity and GSH content were significantly higher (*P* < 0.05) (**Figure 1**). These results document treatment-associated differences in plasma lipid and antioxidant-related biochemical indices.

**Figure 1:**
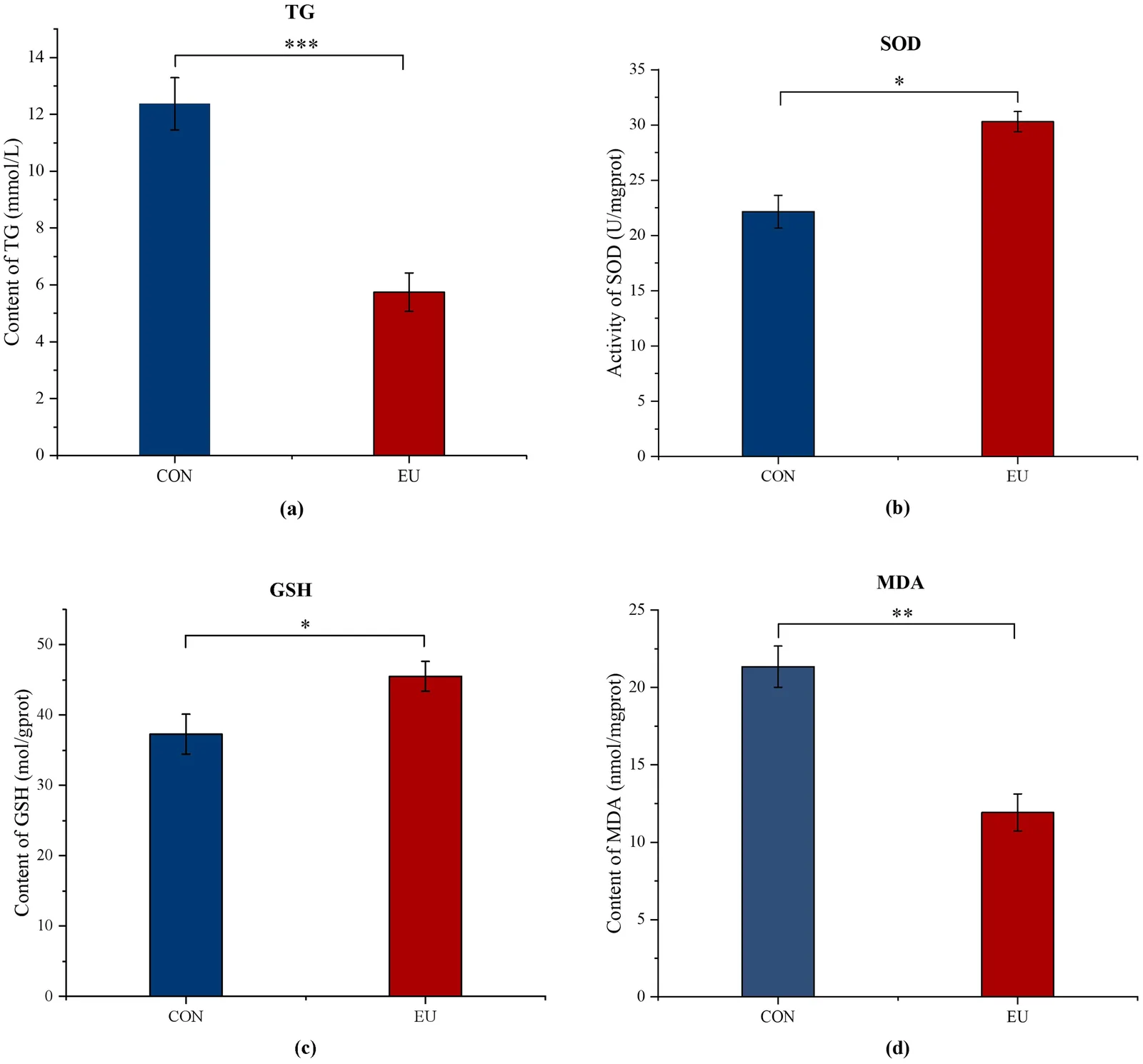
Effects of dietary *E. ulmoides* supplementation on plasma biochemical parameters of *L. crocea*. **(a)** Triglyceride (TG) content; **(b)** superoxide dismutase (SOD) activity; **(c)** glutathione (GSH) content; and **(d)** malondialdehyde (MDA) content. Data are presented as mean ± SD (n = 4). *P* < 0.05, *P < 0.01, and **P < 0.001.

### 3.3 Hepatic lipid deposition assessed by Oil Red O staining

Oil Red O staining revealed extensive red-stained lipid deposits in liver sections from the CON group, whereas the EU group showed visibly less extensive Oil Red O-positive staining and a lower apparent density of lipid droplets (**Figure 2**). This histological pattern was consistent with the lower HSI and plasma TG level observed in the EU group and provides morphological evidence that dietary supplementation with 0.3% E. ulmoides was associated with reduced hepatic lipid deposition. Because the Oil Red O-positive area was not quantified morphometrically, the histological comparison is interpreted qualitatively rather than as a statistically tested difference.

**Figure 2:**
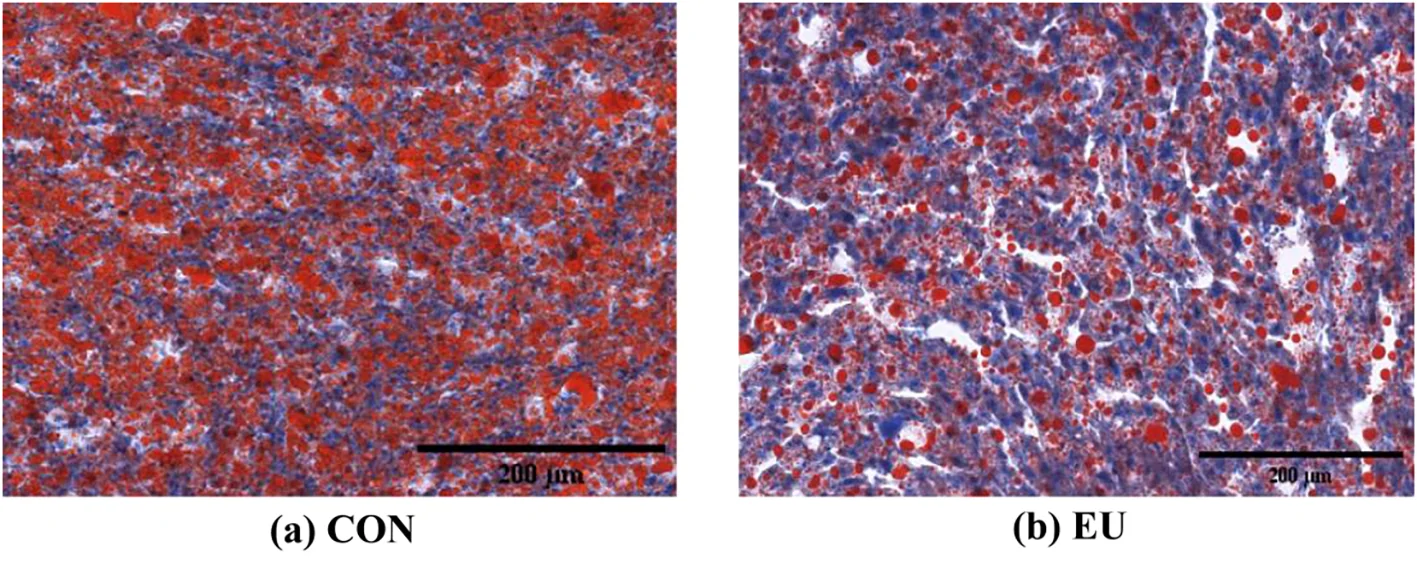
Representative Oil Red O-stained liver sections of *L. crocea* after the 28-day feeding trial. **(a)** CON group fed the basal diet; **(b)** EU group supplemented with 0.3% *E. ulmoides*. Neutral lipids are stained red and nuclei are counterstained blue. Scale bars = 200 μm.

### 3.4 Effect of *E. ulmoides* on the lipidome of *L. crocea*

The OPLS-DA score plot showed tight clustering of samples within each group and clear separation between groups, indicating distinct lipidomic profiles (**Figure 3a**). Permutation testing yielded R² = (0.0, 0.94) and Q² = (0.0, 0.03) (**Figure 3b**).

**Figure 3:**
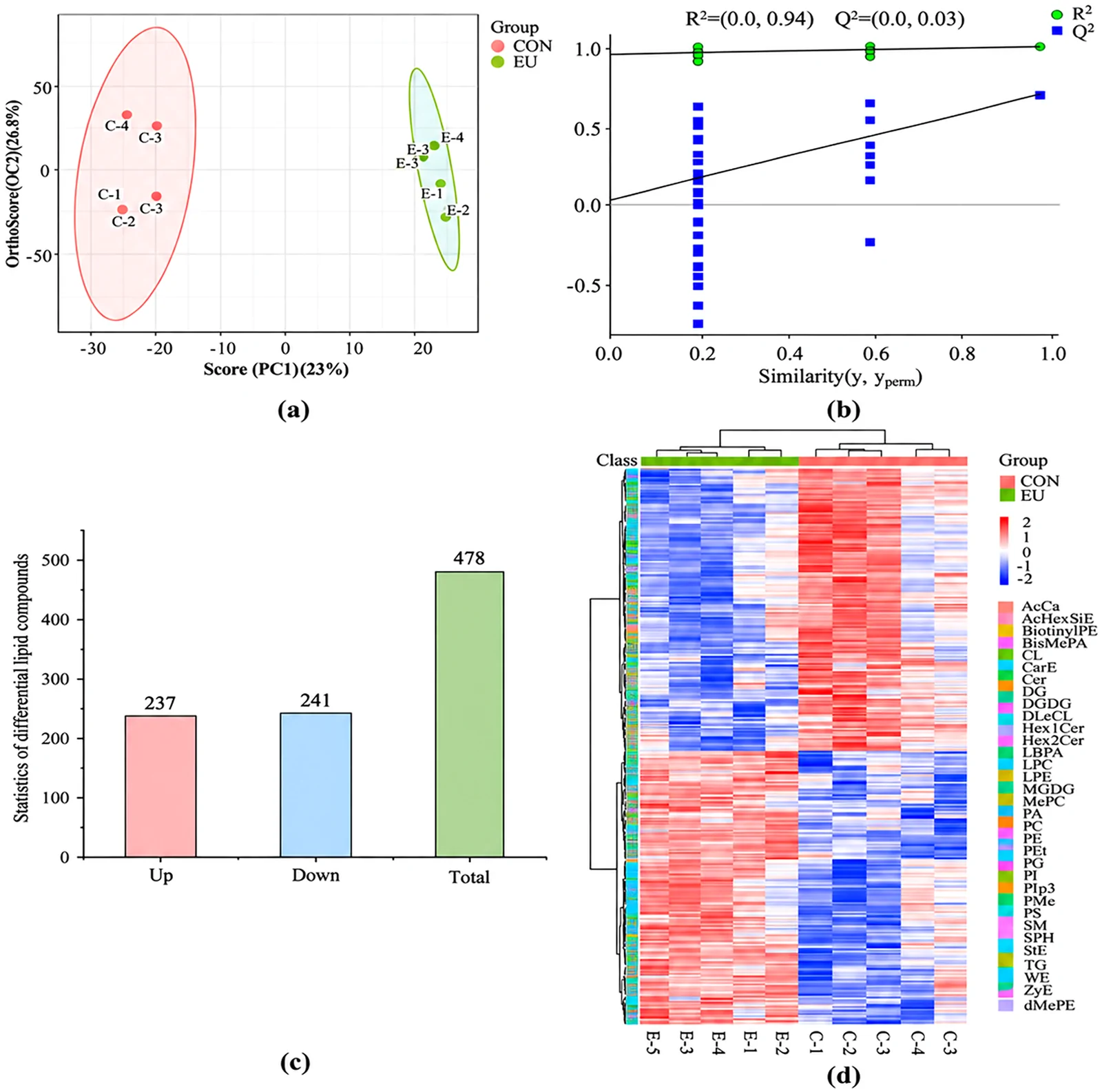
Effects of *E. ulmoides* on the lipidomic profile of *L. crocea* (n = 5 biological replicates per group). **(a)** OPLS-DA score plot showing sample separation between groups; **(b)** OPLS-DA permutation test, with R² and Q² representing model-fit and predictive parameters, respectively; **(c)** numbers of increased and decreased DLMs; and **(d)** heatmap of DLMs, in which red and blue indicate higher and lower relative lipid abundance, respectively.

*E. ulmoides* markedly altered the lipidomic profile of *L. crocea*, with 478 DLMs identified in the EU group compared with the CON group, including 237 upregulated and 241 downregulated lipid molecules (**Figure 3c**). Hierarchical clustering further showed distinct changes in lipid composition between the two groups (**Figure 3d**), involving several major lipid subclasses, particularly glycerides, glycerophospholipids, and sphingolipids. Among the subclasses associated with glycerolipid and glycerophospholipid metabolism, PC contained the largest number of DLMs (102; 63 upregulated and 39 downregulated), followed by TG (90; 25 upregulated and 65 downregulated), PE (38; 29 and 9), DG (30; 17 and 13), PG (22; 7 and 15), PS (16; 5 and 11), CL (15; 10 and 5), and Cer (9; 4 and 5). To further characterize the major molecular changes within these subclasses, representative DLMs were selected using more stringent criteria of |log_2_FC| ≥ 2, *P* < 0.01, and VIP > 1.5, together with their relevance to the glycerolipid and glycerophospholipid metabolic pathways (**Table 3**). Ten representative lipid molecules were retained, including PC(16:1_18:3), PC(22:0_20:5), TG(22:6_14:3_22:6), TG(18:1_11:2_22:6), PE(18:1e), DG(40:3e), PG(38:2e), PS(36:4e), CL(74:8), and Cer(d16:2_25:2). For PC and TG, which contained the largest numbers of DLMs, both upregulated and downregulated molecular species were included to reflect their bidirectional remodeling. These quantitative data complement the global clustering pattern shown in **Figure 2d** and provide representative molecular-level evidence for the major lipid alterations associated with *E. ulmoides* supplementation.

**Table 3:** Representative differential lipid molecules selected from major lipid subclasses in *L. crocea* following dietary *E. ulmoides* supplementation.

Subclass	Representative lipid	Log_2_FC	Regulation
PC	PC(16:1_18:3)	-3.82	Down
PC	PC(22:0_20:5)	3.11	Up
TG	TG(22:6_14:3_22:6)	-4.21	Down
TG	TG(18:1_11:2_22:6)	2.33	Up
PE	PE(18:1e)	-4.40	Down
DG	DG(40:3e)	-2.33	Down
PG	PG(38:2e)	2.15	Up
PS	PS(36:4e)	-2.85	Down
CL	CL(74:8)	-4.94	Down
Cer	Cer(d16:2_25:2)	-3.80	Down

DLMs were subjected to functional enrichment analysis using the LION lipid database (**Table 4**). Significant ontology terms included negative and positive intrinsic curvature, lipid-mediated signaling, ether-bond-containing lipids, polyunsaturated fatty acids, long-chain fatty acids, lysoglycerophospholipids, and lipid species containing C20:5 or C22:5 fatty acyl chains. These enrichment results describe functional and structural annotations associated with the observed DLMs and do not, by themselves, demonstrate changes in membrane function, signaling activity, inflammatory status, or energy metabolism.

**Table 4:** Significantly enriched lipid ontology terms identified by LION analysis in *L. crocea* following dietary *E. ulmoides* supplementation.

LION ID	Description	Hits	*P* value
0000464	negative intrinsic curvature	15	0.0071
0012009	lipid-mediated signaling	10	0.0127
0000467	contains ether-bond	8	0.0241
0002973	fatty acid with 5 double bonds	21	0.007
0002945	fatty acid with more than 18 carbons	59	0.0091
0002967	polyunsaturated fatty acid	59	0.0141
0002977	fatty acid with 3–5 double bonds	42	0.0148
0000021	glycerophosphoglycerophosphoglycerols [GP12]	3	0.0183
0000728	diacylglycerophosphoglycerophosphodiradylglycerols [GP1201]	3	0.0183
0002936	C22:5	10	0.0309
0002976	fatty acid with more than 3 double bonds	50	0.0358
0002961	fatty acid with 22 carbons	26	0.038
0002950	fatty acid with 22–24 carbons	29	0.039
0001741	below average transition temperature	10	0.04
0080971	high bilayer thickness	10	0.044
0000466	positive intrinsic curvature	6	0.046
0000599	lysoglycerophospholipids	6	0.046
0002930	C20:5	7	0.048

### 3.5 Effects of *E. ulmoides* on the transcriptome

Transcriptomic PCA showed clear separation between the two groups (**Figure 4a**). A total of 474 DEGs were identified in the EU group relative to the CON group, including 175 upregulated and 299 downregulated genes (**Figures 4b, c**). Hierarchical clustering also showed distinct transcriptional profiles between groups (**Figure 4d**). KEGG enrichment analysis of the DEGs identified pathways related to amino acid metabolism, lipid metabolism, xenobiotic biodegradation and metabolism, membrane transport, and folding, sorting and degradation among the top enriched categories (**Figure 4e**).

**Figure 4:**
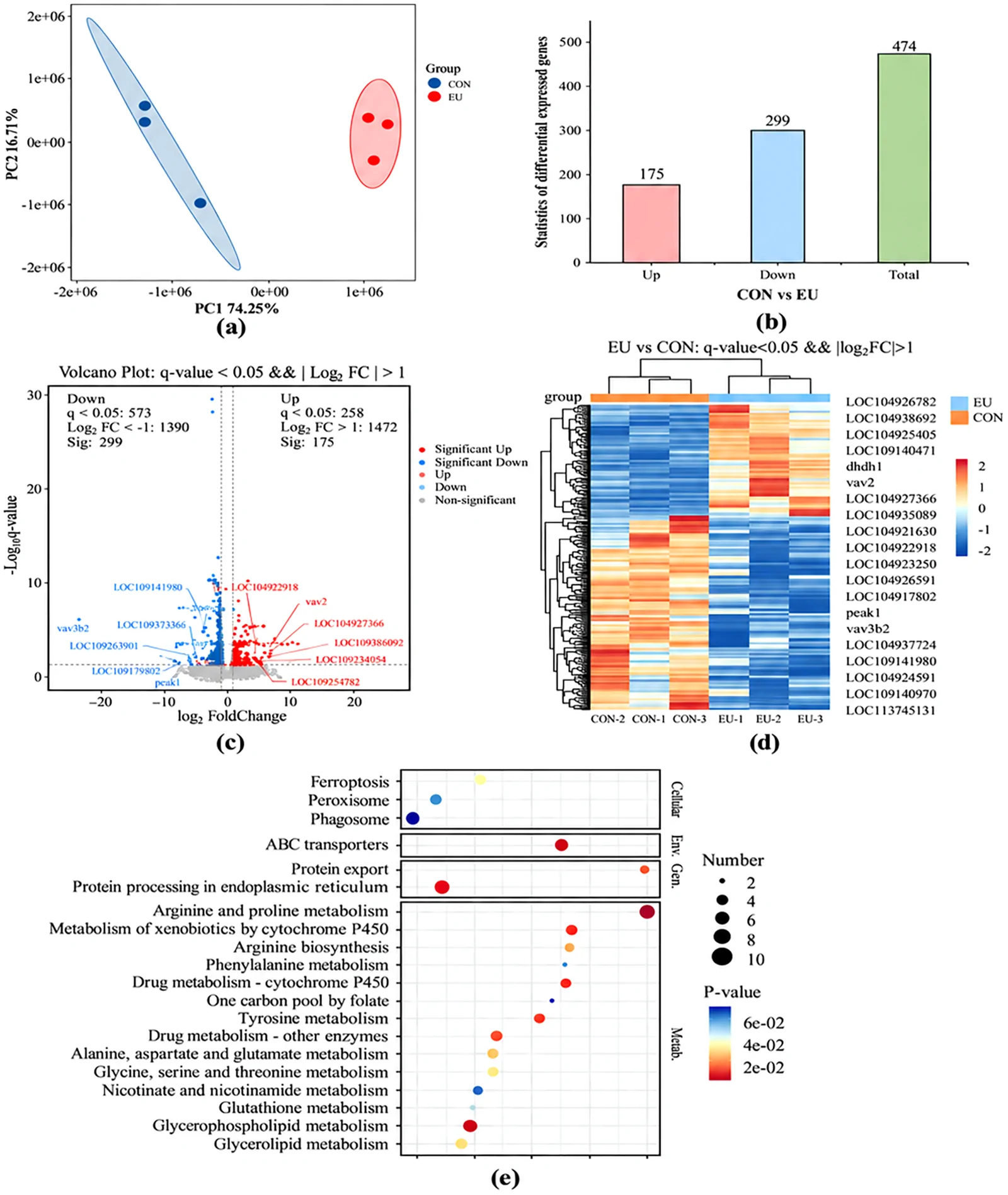
Effects of *E.* ulmoides on the transcriptome of *L.* crocea (n = 3 biological replicates per group): **(a)** PCA score plot; **(b)** bar chart of DEGs; **(c)** volcano plot of DEGs, with upregulated genes shown in red, downregulated genes in blue, and nonsignificant genes in gray; **(d)** heatmap of DEGs, with red and blue indicating higher and lower expression, respectively; and **(e)** bubble plot of the top 20 enriched KEGG pathways.

To provide gene-level interpretation of the enriched pathways, representative DEGs from pathways closely related to the central themes of this study were further examined (**Table 5**). Within lipid metabolism and redox homeostasis, glycerophospholipid metabolism included downregulated *pcyt2* and upregulated *lpgat1*, *gpam*, *dgki*, and *pla2g10*, whereas glycerolipid metabolism included upregulated *gpam*, *dgki*, and *agpat2* together with downregulated *aldh3a*. Glutathione metabolism was represented by downregulated *mgst1*, *gpx4*, and *gstt1a*. Several amino-acid-related pathways also showed coordinated transcriptional changes: arginine and proline metabolism contained downregulated *agmat*, *prodh*, and *arg1* and upregulated *odc1*; arginine biosynthesis contained downregulated otc, *arg1*, and *gpt2*; tyrosine metabolism contained downregulated *hpda* and *ddc*; alanine, aspartate and glutamate metabolism contained downregulated *gpt2* and upregulated *cad*; and glycine, serine and threonine metabolism contained downregulated *bhmt* and *alas2*. In addition, the ABC transporter pathway included upregulated *abca3*, *abcd2*, and *abcd4*, whereas representative genes involved in protein export (*sec61g*, *sec11a*, and *spcs3*) and protein processing in the endoplasmic reticulum (*hsp90b1*, *pdia4*, and *calr3b*) were downregulated.

**Table 5:** Representative DEGs in KEGG pathways relevant to the metabolic responses of *L. crocea* to dietary *E. ulmoides* supplementation.

Category	Pathway definition	Gene id	Gene name	Regulation
Lipid metabolism	Glycerophospholipid metabolism	LOC104930254	*pcyt2*	down
		LOC104931239	*lpgat1*	up
		LOC104936714	*gpam*	up
		LOC104936319	*dgki*	up
		LOC104918718	*pla2g10*	up
	Glycerolipid metabolism	LOC104936714	*gpam*	up
		LOC104936319	*dgki*	up
		LOC104929186	*agpat2*	up
		LOC104930668	*aldh3a*	down
	Glutathione metabolism	LOC104937933	*mgst1*	down
		LOC104930512	*gpx4*	down
		LOC104934872	*gstt1a*	down
Amino acid metabolism	Arginine and proline metabolism	LOC104928636	*agmat*	down
		LOC104923327	*odc1*	up
		LOC104924608	*prodh*	down
		LOC104938905	*arg1*	down
	Arginine biosynthesis	LOC104930898	*otc*	down
		LOC104938905	*arg1*	down
		LOC104917713	*gpt2*	down
	Tyrosine metabolism	LOC104928774	*hpda*	down
		LOC104939491	*ddc*	down
	Alanine, aspartate and glutamate metabolism	LOC104917713	*gpt2*	down
		LOC104928828	*cad*	up
	Glycine, serine and threonine metabolism	LOC104930138	*bhmt*	down
		LOC104920213	*alas2*	down
Membrane transport and protein processing	ABC transporters	LOC104925721	*abca3*	up
		LOC104931543	*abcd2*	up
		LOC104930542	*abcd4*	up
	Protein export	LOC104922587	*sec61g*	down
		LOC104940662	*sec11a*	down
		LOC104933675	*spcs3*	down
	Protein processing in endoplasmic reticulum	LOC104940626	*hsp90b1*	down
		LOC104922362	*pdia4*	down
		LOC104931427	*calr3b*	down

### 3.6 RT-qPCR

Based on the KEGG pathway enrichment analysis, four DEGs were selected from glycerolipid metabolism pathway and glycerophospholipid metabolism pathway, which was the key pathway closely related to lipid metabolism. The relative expression levels of 4 DEGs, including *aldh*, *dgki*, *gpam*, and *lpgat1*, were measured by RT-qPCR (**Figure 5**). The expression trends observed in RT-qPCR were consistent with those obtained from RNA-seq analysis, indicating the overall reliability and reproducibility of the transcriptome sequencing data.

**Figure 5:**
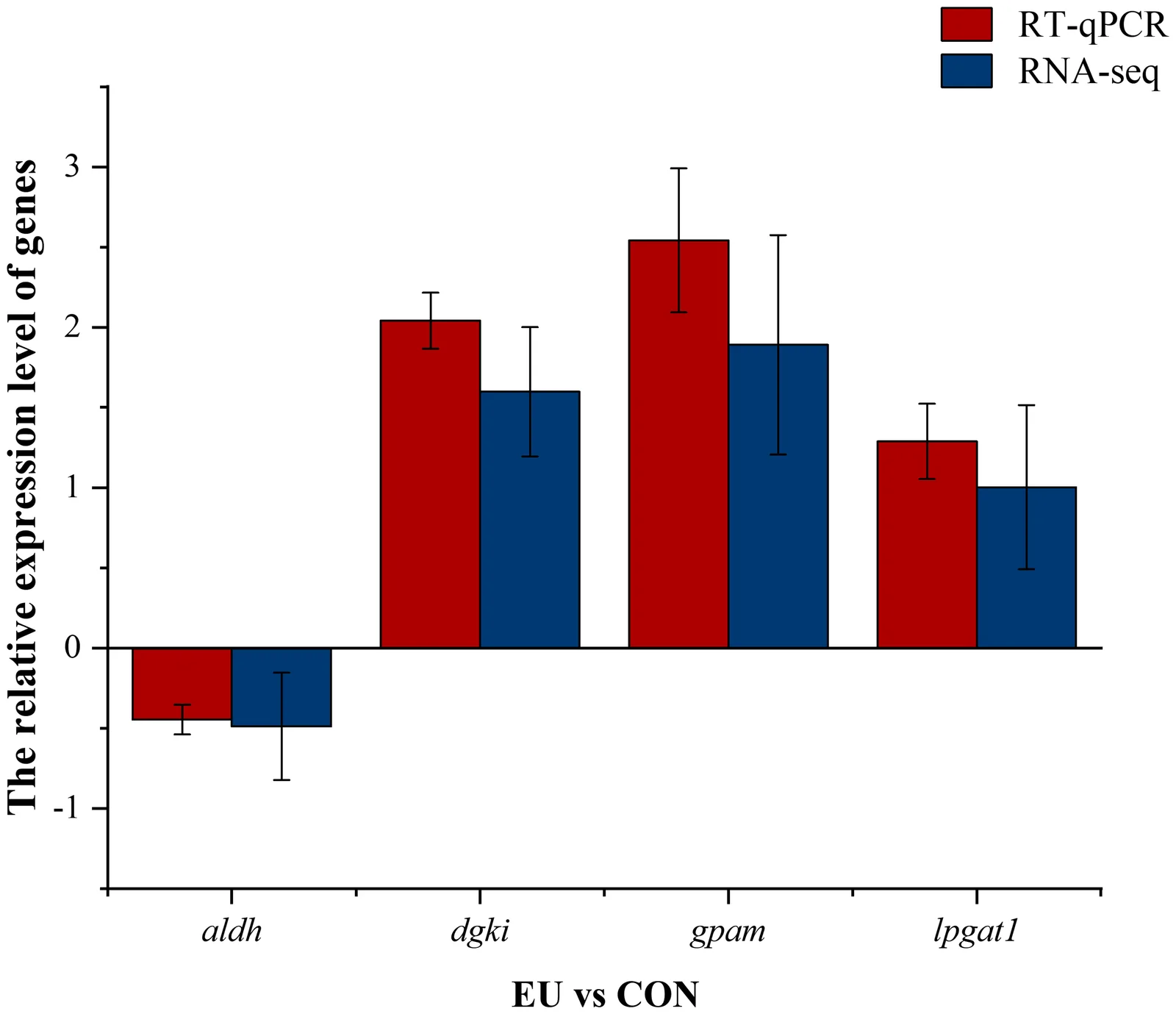
Validation of DEGs using RT-qPCR.

### 3.7 Integrated analysis of transcriptome and lipids

Based on the transcriptomic and lipidomic results, DEGs and DLMs were jointly mapped onto KEGG pathways. Glycerophospholipid metabolism and glycerolipid metabolism were the principal pathways represented in both omics datasets (**Figure 6**). DEGs mapped to glycerolipid metabolism were associated with reactions involving intermediate metabolites related to neutral lipid synthesis, whereas DEGs mapped to glycerophospholipid metabolism were associated with reactions involving PG, PC, PE, and their precursors. The two pathways converged at phosphatidic acid (PA; 1,2-diacyl-sn-glycerol-3-phosphate) in the KEGG pathway topology. Importantly, PA was identified here as a shared pathway intermediate rather than as a directly quantified differential lipid molecule. Thus, the integrated analysis demonstrates convergence of transcriptomic and lipidomic alterations at the pathway level but does not establish direct statistical co-regulation between individual DEGs and DLMs.

**Figure 6:**
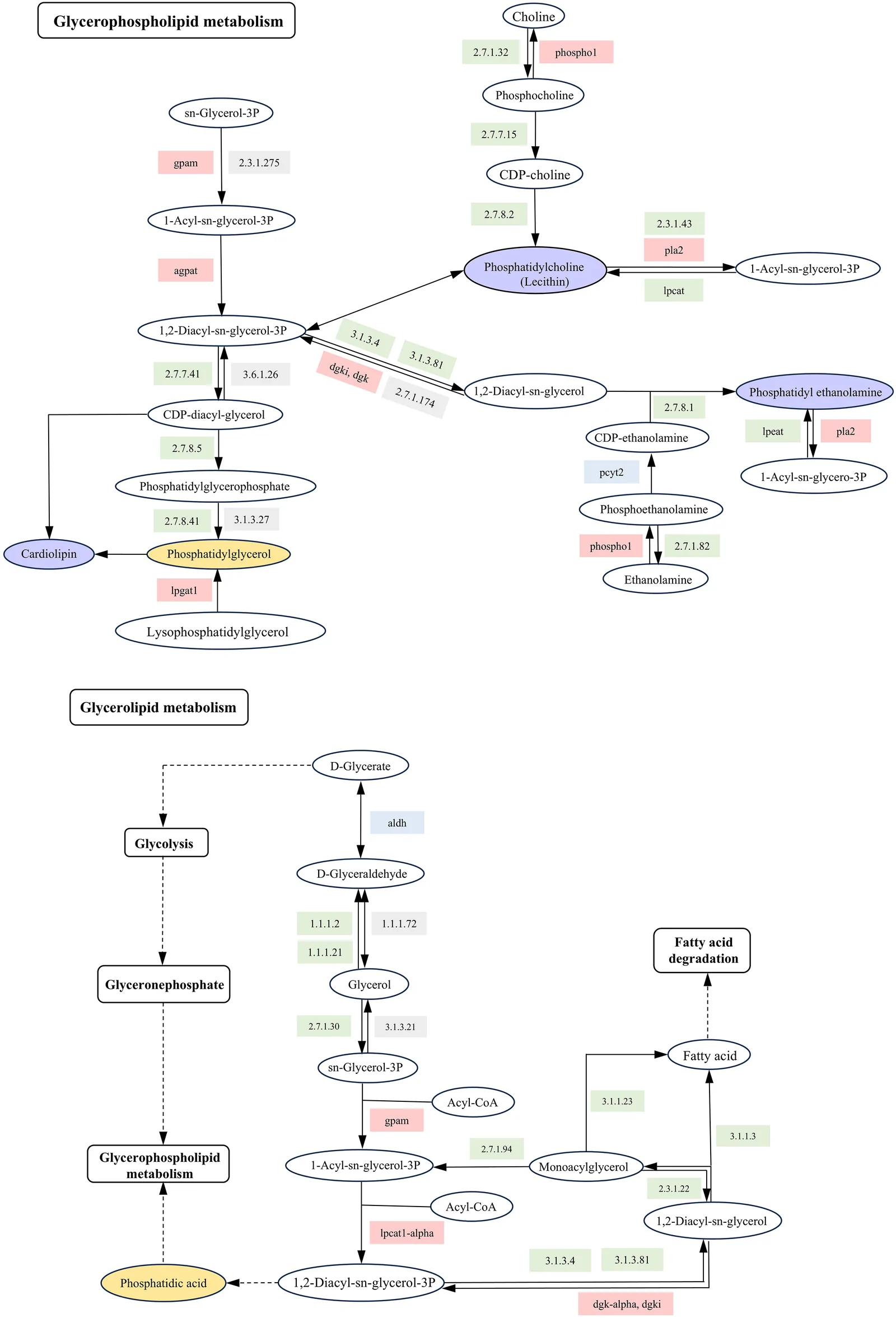
Pathway map of combined omics analysis. Boxes represent transcriptome genes, with pink and blue indicating upregulated and downregulated DEGs, respectively. Ellipses represent metabolites, with yellow and purple indicating upregulated and downregulated DLMs in the lipidomic data, respectively.

## 4 Discussion

*E. ulmoides* is a Chinese herbal medicine with medicinal and edible functions. The addition of 0.3% *E. ulmoides* to feed significantly improved the growth performance of *L. crocea*. *E. ulmoides* significantly reduced HSI and CF values, and Oil Red O staining showed less extensive hepatic lipid staining in the EU group, together supporting an association between dietary *E. ulmoides* supplementation and reduced hepatic lipid deposition. Meanwhile, SGR was significantly elevated, and FCR was significant reduced in the EU group, suggesting E. ulmoides enhanced feed efficiency in *L. crocea*. This conclusion was similar to the report in other aquatic species (Yang et al., 2024; Zhang et al., 2019). Importantly, the concurrent improvement in growth performance and the remodeling of lipid-related biochemical and omics profiles indicates an association between these responses, but does not establish that lipid metabolic remodeling directly caused the growth improvement. Fish growth is a multifactorial trait influenced by nutrient intake, digestion and absorption, amino-acid and protein metabolism, endocrine regulation, and energy partitioning. Because several of these processes were not directly quantified in the present study, the lipid metabolic changes observed here should be regarded as one component of the broader physiological response to dietary *E. ulmoides* rather than as a sole or proven driver of growth. For the plasma biochemical parameters of *L. crocea*, the change of TG content, SOD activity, GSH and MDA levels indicated that *E. ulmoides* help prevent hyperlipidemia, enhance the antioxidant capacity, and reduce oxidative damage.

Lipids contain important biomolecules that regulate various life activities, including energy conversion, material transport, signal recognition and transduction, cell differentiation, and apoptosis (Jeon et al., 2023). In this study, we systematically analysed the regulatory effects of *E. ulmoides* on the lipid composition and related biological functions of *L. crocea* using lipidomics technology. On the one hand, *E. ulmoides* significantly influenced the physical properties of the cell membrane, including curvature, thickness, and fluidity (**Table 3**). These changes enhanced the stability of the cell membrane through modulation of the lipid composition, and this adaptation was further strengthened by regulating membrane-associated signaling molecules (PC, PE, Cer), which facilitated lipid-mediated signaling to maintain and optimize cellular functions (Li et al., 2006). On the other hand, ether phospholipids and polyunsaturated fatty acids (PUFAs) were significantly enriched. These lipids are critical for antioxidant defense, which could preserve membrane integrity and reduce lipid peroxidation induced by oxidative stress (Cui et al., 2021). In addition, LION analysis revealed enrichment of lipid species containing C20:5 and C22:5 fatty acyl chains, indicating remodeling of LC-PUFA-associated lipid composition following dietary *E. ulmoides* supplementation. These fatty acyl chains are commonly associated with n-3 LC-PUFA metabolism and may contribute to membrane organization, lipid signaling, and inflammatory homeostasis (Ishibashi et al., 2021). PUFAs and their metabolites can modulate inflammatory responses through signaling pathways involving transcription factors such as peroxisome proliferator-activated receptors (PPARs) and nuclear factor-κB (NF-κB) (Bazinet and Layé, 2014). Thus, the observed enrichment of C20:5- and C22:5-containing lipid species suggests that *E. ulmoides* supplementation may influence inflammatory homeostasis through LC-PUFA-associated lipid remodeling, although this possibility requires further experimental validation. Furthermore, *E. ulmoides* may significantly affect the expression of lipids related to energy metabolism, including TG and DG, thereby optimizing energy utilization and enhancing adaptability of fish under high-density culture conditions.

In addition to lipid metabolism, KEGG analysis also reveals significant enrichment of pathways such as arginine and proline metabolism, ABC transporters, and protein export. Amino acid metabolism is closely associated with fish growth, as amino acids not only serve as substrates for protein synthesis but also act as important energy sources during the juvenile fish stage (Kaushik and Seiliez, 2010). Arginine and proline are involved in nitrogen metabolism and collagen-related metabolic processes (Karna et al., 2020; Wu et al., 2009). Moreover, previous studies have demonstrated that the addition of *E. ulmoides* in feed can influence physiological processes related to collagen metabolism in the muscles of *C. idellus* (Yang et al., 2022). In this study, the arginine and proline metabolism pathway exhibits obvious transcriptional remodeling, with the down-regulation of *agmat*, *prodh*, and *arg1*, and the up-regulation of *odc1*, indicating that this pathway is affected by *E. ulmoides* treatment. However, this mixed expression pattern does not directly prove enhanced protein synthesis or collagen deposition. Meanwhile, the up-regulation of *abca3*, *abcd2*, and *abcd4* in the ABC transporter pathway, along with the differential expression of related genes in the protein export pathway, suggests transcriptional adjustments in processes related to cellular material transport and protein processing. Considering that ABC transporters can participate in the transmembrane transport of various lipids, metabolites, and cofactors (Akhtar and Turner, 2022; Tarling et al., 2013), and the protein export pathway involves the transmembrane transport, processing, and secretion of nascent proteins, these changes may reflect a broader nutritional metabolism and cellular homeostasis adaptation induced by *E. ulmoides*. However, this study did not directly measure muscle protein content, amino acid deposition, collagen content, or protein synthesis rate. Therefore, it cannot be inferred that these pathways directly lead to increased muscle protein deposition or accelerated growth. A more reasonable explanation is that the transcriptional changes at the pathway level occur concomitantly with the growth-improvement phenotype observed in this study and may constitute part of the overall metabolic adaptation induced by *E. ulmoides*. Their specific physiological contributions still need to be further verified through muscle composition analysis, amino acid deposition measurement, and protein synthesis-related indicators. In addition, the xenobiotic metabolism pathway mediated by cytochrome P450 is also significantly enriched. Cytochrome P450 (CYP) enzymes is involved in the oxidation and clearance of exogenous compounds and plays an important regulatory role in endogenous metabolic homeostasis (Zhao et al., 2021), suggesting that *E. ulmoides* treatment may also affect the adaptability of *L. crocea* to exogenous chemical stimuli and metabolic load. Overall, these enriched pathways indicate that, in addition to influencing lipid metabolism, *E. ulmoides* is accompanied by transcriptional remodeling of multiple processes such as amino acid metabolism, cellular material transport, protein processing, and xenobiotic metabolism, providing a potential molecular background for its improvement of the overall metabolic state and growth performance of large yellow croaker. However, the direct causal relationship between these pathways and growth still awaits further experimental verification.

In this study, the integrated omics analysis identified glycerolipid metabolism and glycerophospholipid metabolism as the principal lipid metabolic pathways represented in both datasets. These pathways have distinct major functions, with glycerolipid metabolism contributing to neutral-lipid synthesis and storage (Hagopian et al., 2008; Scott et al., 2014) and glycerophospholipid metabolism contributing to membrane phospholipid synthesis and remodeling (Dong et al., 2023; Morita and Ikeda, 2022; Van der Veen et al., 2017), but they also share metabolic intermediates. Within glycerolipid metabolism, *gpam*, *agpat2*, and *dgki* were upregulated. *gpam* and *agpat2* participate in sequential acylation reactions of glycerol-3-phosphate-derived intermediates toward phosphatidic acid (PA), whereas DGKI catalyzes the phosphorylation of diacylglycerol (DAG) to PA. These transcriptional changes suggest that reactions surrounding the glycerol-3-phosphate-PA and DAG-PA nodes may be altered following dietary *E. ulmoides* supplementation. Importantly, PA abundance was not directly quantified as a differential lipid molecule in the present lipidomic dataset. Therefore, PA is interpreted here as a putative metabolic node inferred from KEGG pathway topology rather than as an experimentally demonstrated increase in PA abundance. Because PA is a recognized branch-point intermediate between glycerolipid and glycerophospholipid metabolism (Lutkewitte and Finck., 2020), the transcriptional changes around this node, together with pathway-level co-mapping of DEGs and DLMs, support the hypothesis that PA may participate in linking the two pathways. In glycerophospholipid metabolism, the upregulation of *lpgat1* is consistent remodeling of PG biosynthesis, whereas the downregulation of *pcyt2* may indicate altered PE biosynthetic capacity. These transcriptional responses are also consistent with the observed remodeling of glycerophospholipid classes, including PG, PC, PE, and CL, although changes in transcript abundance do not directly establish corresponding changes in enzymatic activity or metabolic flux. Future studies should directly quantify PA molecular species by targeted lipidomics and assess metabolic flux, together with protein-level or enzymatic validation of *gpam*, *agpat2*, *dgki*, *lpgat1*, and *pcyt2*, to determine whether PA-mediated pathway coupling contributes to the lipid metabolic response to *E. ulmoides*. Future studies should directly quantify PA molecular species by targeted lipidomics and assess metabolic flux, together with *in vitro* functional assays and protein-level/enzymatic validation of *gpam*, *agpat2*, *dgki*, *lpgat1*, and *pcyt2*, to determine whether the observed transcriptional changes translate into altered catalytic function and whether PA-mediated pathway coupling contributes to the lipid metabolic response to *E. ulmoides*.

The present study provides molecular evidence for metabolic responses associated with the improved growth performance of *L. crocea* following dietary *E. ulmoides*, but it does not establish a direct causal mechanism linking lipid metabolic remodeling to growth enhancement. Growth is influenced by multiple interacting processes, and digestive efficiency, nutrient absorption, endocrine regulation, and whole-body energy partitioning were not directly evaluated in this study. These factors should therefore be incorporated into future experiments designed to test the causal contribution of lipid metabolic remodeling to growth. The gut-liver axis represents another testable hypothesis that warrants direct investigation. Future studies should characterize gut microbiota composition and intestinal barrier integrity, including tight-junction-related markers (e.g., occludin, claudins, and ZO-1) and goblet-cell abundance/mucus status, and integrate these measurements with hepatic and systemic metabolic phenotypes to determine whether intestinal responses contribute to the effects associated with *E. ulmoides* supplementation. Accordingly, the present multi-omics findings should be regarded primarily as a hypothesis-generating framework rather than as definitive evidence of a causal mechanism. In addition, this study only designed one concentration group and one experimental time period, and the most appropriate dosage and feeding cycle of the *E. ulmoides* were important subjects that required further research in the future. In addition, the EU diet was not independently analyzed for proximate composition or gross energy; therefore, small treatment-related differences in nutrient composition cannot be completely excluded.

## 5 Conclusions

In conclusion, this study analysed the physiological and molecular responses of *L. crocea* following dietary supplementation with *E. ulmoides*, showing improved growth performance together with marked remodeling of lipid metabolism. Integrated transcriptomic and lipidomic analyses identified glycerophospholipid and glycerolipid metabolism as the principal lipid metabolic pathways represented in both omics datasets. The concurrent occurrence of growth improvement and lipid metabolic remodeling supports an association between these responses, but does not demonstrate a direct causal relationship. These findings provide a scientific basis for further evaluating *E. ulmoides* as a functional feed additive and for experimentally testing how lipid metabolism interacts with other determinants of growth in *L. crocea*. Accordingly, the current multi-omics results should be interpreted primarily as hypothesis-generating evidence. Future work combining *in vitro*/enzymatic validation of *gpam*, *agpat2*, *dgki*, *lpgat1*, and *pcyt2* with targeted PA quantification and metabolic-flux analysis, together with gut-liver axis analyses of the gut microbiota and intestinal barrier features (including tight-junction markers and goblet cells), will be necessary to test the proposed mechanisms.

## Data Availability

The original contributions presented in the study are included in the article and its **Supplementary Material**. Further inquiries can be directed to the corresponding authors.
