# Supplementary material for: Integrative transcriptomic and lipidomic analysis of the effects of Eucommia ulmoides on growth performance and lipid metabolism in Larimichthys crocea
Source: Front Cell Infect Microbiol. 2026 Sep 30;16:1890262. doi: 10.3389/fcimb.2026.1890262 (PMC13661303; doi:10.3389/fcimb.2026.1890262)
Supplement: Supplementary file 1 [file Table1.docx]

**Supplementary data**

**S1. Primer sequence of RT-qPCR**

| **Gene name** | **Forward primer** | **Reverse primer** |
| --- | --- | --- |
| *aldh* | AACTGCACCTGCTGATGTCG | ACTGCAGGTTCCTTCGTTCA |
| *dgki* | TTACACCAGGCGCCATCTAC | TCGATGCGATAGAAGCGGTC |
| *gpam* | GGACACACAAGCGGGACTAAA | CTGCGGTCCGAGTCATCATT |
| *lpgat1* | AGGACCGGTGTGTATCAGGA | ACGGGATGGCTACACAGTTG |
